# Astaxanthin and other Nutrients from *Haematococcus pluvialis*—Multifunctional Applications

**DOI:** 10.3390/md18090459

**Published:** 2020-09-07

**Authors:** Malwina Mularczyk, Izabela Michalak, Krzysztof Marycz

**Affiliations:** 1 Department of Experimental Biology, Faculty of Biology and Animal Science, Wroclaw University of Environmental and Life Sciences, ul. Norwida 27B, 50-375 Wroclaw, Poland; krzysztof.marycz@upwr.edu.pl; 2 Department of Advanced Material Technologies, Faculty of Chemistry, Wrocław University of Science and Technology, Smoluchowskiego 25, 50-372 Wrocław, Poland; izabela.michalak@pwr.edu.pl; 3 International Institute of Translational Medicine, Malin, Jesionowa 11, 55-114 Wisznia Mała, Poland

**Keywords:** *Haematococcus pluvialis*, microalgae, astaxanthin, applications

## Abstract

Bioactive compounds of natural origin are gaining increasing popularity. High biological activity and bioavailability, beneficial effects on health and safety of use are some of their most desirable features. Low production and processing costs render them even more attractive. Microorganisms have been used in the food, medicinal, cosmetic and energy industries for years. Among them, microalgae have proved to be an invaluable source of beneficial compounds. *Haematococcus pluvialis* is known as the richest source of natural carotenoid called astaxanthin. In this paper, we focus on the cultivation methods of this green microalga, its chemical composition, extraction of astaxanthin and analysis of its antioxidant, anti-inflammatory, anti–diabetic and anticancer activities. *H. pluvialis*, as well as astaxanthin can be used not only for the treatment of human and animal diseases, but also as a valuable component of diet and feed.

## 1. Introduction

Nowadays microalgae are gaining in popularity not only because of their high nutrient content, but also because of the promotion of a healthy diet [1]. The employment of microalgae on an industrial scale began in 1950, when Burlew proposed the use of microalgae as an alternative source of protein for plants and animals. Since then, algae cultivation has become more common, not only for the application in the food industry, but also for animal and aquaculture feed purposes [2,3,4,5]. Microalgae, as a source of active biomolecules, are used in the pharmaceutical and cosmetics industry [2,3]. Algae are gaining also a lot of attention in terms of the production of biodegradable plastics and biofuels [2,6,7].

The application of microalgae on an industrial scale is facilitated by the rapid growth of biomass, the conversion of CO_2_ in the process of photosynthesis, not requiring environmental conditions, and huge demand in food and energy production [8]. Microalgae can occur in oceans, rivers or lakes [9]. *Haematococcus pluvialis* can grow in particularly difficult conditions, such as the arctic waters of the White Sea [6].

Researchers are continuously confirming the beneficial effects of microalgae components and their biological properties (e.g., antioxidant and anti-inflammatory) [10]. Among those components, starch, cellulose and β-1,3 glucan are considered as the most important polysaccharides. These substances are used in the pharmaceutical and cosmetics industries as well as in dietary supplements [3,5,11]. The same goes for lipids such as hydrocarbons and polyunsaturated fatty acids. Many of the bioactive compounds which have strong antibacterial, antifungal and antiviral properties have found application in the production of vaccines, antibiotics, agrochemicals and cosmetics [12]. Proteins such as enzymes, amino acids and polypeptides are used in the food and pharmaceutical industries, whereas pigments including carotenoids or chlorophylls are widely employed in the food technology, chemical and pharmaceutical industries [13]. 

**Figure 1:**
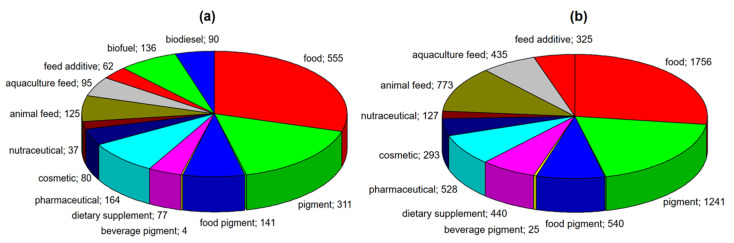
Potential applications of (**a**) *Haematococcus pluvialis* and (**b**) astaxanthin, extracted from this microalga (according to the Web of Science, accessed on 3 August, 2020).

Among many species of microalgae, one of the most important is *Haematococcus pluvialis*, which is a source of one of the strongest natural antioxidants, which is astaxanthin [14]. This microalga has very wide applications, which are shown in Figure 1a. This figure summarizes the number of publications describing the potential use of *H. pluvialis* (Figure 1a), as well as its high-value molecule-astaxanthin (Figure 1b), according to the Web of Science database. 

**Figure 2:**
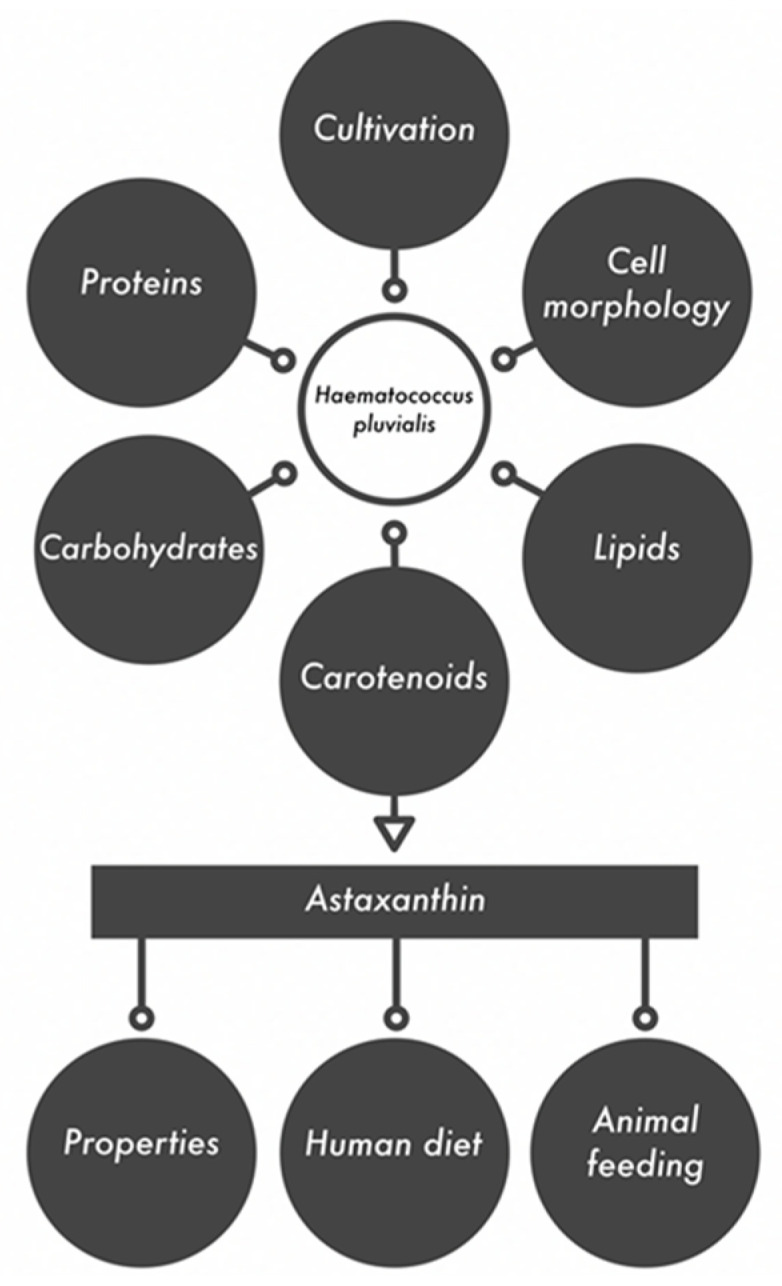
*Haematococcus pluvialis* as a source of active compounds and their applications.

Most applications of both microalga and astaxanthin are related to human nutrition (food, pigments for food and beverages) and health (pharmaceuticals, nutraceuticals and dietary supplements). Much fewer publications are devoted to the use of microalga and extracted astaxanthin in animal nutrition. Therefore, in the presented review, we focused on the biological properties of astaxanthin (antioxidant, anti-inflammatory and anti–diabetic, anticancer) and its application in the nutrition and treatment of humans and animals. These descriptions were preceded by information on the cultivation of *Haematococcus pluvialis*, its biochemical composition and isolation of astaxanthin, also by means of the innovative extraction techniques. This review presents a comprehensive approach to astaxanthin. The general outline of this review is presented in Figure 2.

## 2. Cell Morphology of Haematococcus Pluvialis

**Figure 3:**
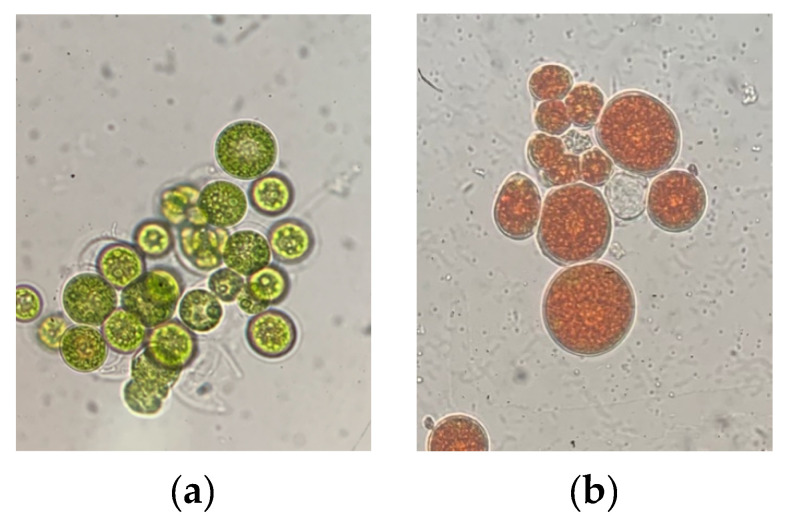
Cell morphology of *Haematococcus pluvialis* on (**a**) green stage (**b**) red stage (own source).

*Haematoccocus pluvialis* is a unicellular, spherical, green biflagellate oleaginous cell with a diameter of ~30 μm [15,16]. Based on their life cycle, morphology and physiology, *H. pluvialis* can either exist as vegetative green cells (Figure 3a)—capable of swimming due to their two flagellas, closely connected with green stage and biomass accumulation—or as red cysts (Figure 3b), which accumulate astaxanthin in response to stressful environmental conditions. The cells observed during the vegetative phase are: macrozooids (zoospores), microzooids and palmella, while the cells of the astaxanthin accumulation phase are asexual aplanospores [17].

Macrozooids are biflagelled, spherical, ellipsoidal or pear-shaped cells with dimensions of 8 to 20 µm. Cells at this stage can produce 2–32 daughter cells by asexual reproduction, through the formation of a sporangium [18]. Unfavourable conditions can cause an increase in cell size and loss of both flagellas. During that process, within 1–2 days, macrozoids become an amorphous, multilayered, non-motile form called palmella. Persisting unfavourable conditions lead to the transformation into the asexual aplanospore stage [19]. A thick and rigid trilaminar sheath and the secondary cell wall protects aplanospores against acetolysis, high light irradiance and high salinity. Accumulation of astaxanthin usually occurs in aplanospores and is induced by nutrient deprivation or high light exposure [20]. Numerous studies have been performed in order to assess the effect of nitrogen limitation or light intensity on cell morphology. The obtained collective information enables a more accurate optimization of the *H. pluvialis* culture process. More detailed studies on the correlation between cell structure in each growth phase and the mechanisms of astaxanthin accumulation could further improve the production of expected metabolites.

## 3. Cultivation of Haematococcus Pluvialis

Optimized environmental parameters allow for achieving high biomass growth and, consequently, astaxanthin accumulation. Cultivation of *Haematococcus pluvias* can be divided into two stages: the first one, called green stage, and the second one–red stage [21]. At the beginning, microalgae divide into daughter cells under favorable temperature, pH, amounts of nitrates, metals or a light wave length and intensity. In response to adverse conditions, cells typically stop dividing. Stress conditions cause accumulation of carotenoids, mostly astaxanthin. The temperature during the green phase should be within a range of 25–30 °C [22]. The light intensity below 150 µmoL photons m^−2^ s^−1^ enables cell division. The typical irradiation is 40–50 μmoL photons m^−2^ s^−1^ and pH should be kept at 7 [23]. Cycles of alternating light and darkness are 12:12 or 16:8 [24]. The most commonly used media for *H. pluvialis* growth are KM1, BBM, Z8, BG- 11, OHM, and their modified versions. BBM and Z8 are used for autotrophic cultures while KM1 medium is suitable for heterotrophic cultures. A mixture of BBM medium and an organic carbon source, e.g., sodium acetate, can be used in a mixotrophic culture [25,26]. There are several growth modes of microalgae. In a photoautotrophic mode, microalgae use light as a source of energy and an inorganic compound as a source of carbon; in that case, light is obligatory. For the photoheterotrophic mode, however, the source of carbon is organic and light is not obligatory but it might be used aa a source of energy. In a heterotrophic mode, a source of energy and carbon are both organic. The mixotrophic way of microalgae cultivation uses organic and inorganic sources of carbon and energy [27]. After reaching a high cell concentration, the red phase (the astaxanthin accumulation phase) occurs. For this purpose, stress factors are used: hunger for nitrogen, high light intensity, high temperature or the presence of metals [28]. 

For mass production of *H. pluvialis*, bioreactors or production ponds are used, it is also possible to combine both systems. The main factors in selecting a cultivation method are: capital and operational costs, cultivation area, climatic conditions (light, temperature, rain), the possibility of contamination, water availability, level of automation and system efficiency [29]. Many producers of natural astaxanthin belong to the association of producers of natural astaxanthin derived from *Haematococcus pluvialis*: the Natural Algae Astaxanthin Association (NAXA) [30]. Cyanotech Corporation in Kailua-Kona, Hawaii, is one of the producers of natural astaxanthin from *Haematococcus*. The large 500-cubic-meter ponds of algal culture have an annual production capacity of more than 70 metric tons of *Haematococcus* algae meal with a minimum of 1.5% of astaxanthin (NatuRose^®^) [31]. Mera Pharmaceuticals, Cyanotech Inc., Algatechnologies Ltd., Biogenic Co. Ltd. are some of the more important producers of *H. pluvialis*-derived astaxanthin. Over 95% of astaxanthin available on the market is chemically synthesized, only < 1% is obtained from *H. pluvialis* [7].

To this date, many laboratory scale *H. pluvialis* cultivation models were considered. In the Web of Science database (accessed 3 August 2020), over 392 publications concern methods of *H. pluvialis* cultivation. However, the industrial scale is of greater interest. Microbial infection of the culture and uncompetitive costs of obtaining astaxanthin, relative to chemical synthesis are the main obstacles to overcome. The optimization of *H. pluvialis* cultivation could contribute to the creation of local microalgae crops for astaxanthin production. The use of municipal and industrial waste substrates for industrial cultivation would increase the attractiveness of *H. pluvialis*, while reducing the costs of the process. However, *H. pluvilais* has the ability to absorb metal ions in bioremediation processes and poses a risk of heavy metal contamination when cultivated in wastewater. Therefore, it is necessary to either control the quality of waste or thoroughly purify astaxanthin and other metabolites before introducing them into the human or animal diet [32].

## 4. Biochemical Composition of *Haematococcus Pluvialis*

### 4.1. Proteins and Carbohydrates

The maturation of cells and the passage through successive stages of life cycles results in an altered biochemical profile of the cell. Most of *H. pluvialis* green stage cells are characterized by a high protein content of 29%–45% per dry weight (d.w.) [33]. Protein content in palmella decreases to 36% d.w., while in the red stage cells, this content is within a range of 21%–23% d.w. Carbohydrates as starch allow the cell to survive during a prolonged stress. The content increases from 15%–17% d.w. in the green stage to 60%–74% d.w. in the red cyst. As Recht et al. (2012) demonstrated in their research, total carbohydrates can increase rapidly, by up to 63% d.w, during the first day of stress exposure, decrease to 41% d.w. on the following day, and remain at this level until the end of the cultivation [34,35]. 

### 4.2. Lipids

In the green stage, total lipid content varies from 20% to 25% (Table 1), with approximately 10% of the lipids being composed predominantly of polyunsaturated fatty acids (PUFAs) deposited in chloroplasts [14]. The accumulation of lipids ensues from unfavorable environmental conditions. These include limiting nitrogen and phosphorus content, high salinity, high light intensity and extreme temperatures. According to Hagen et al. (2002), the most effective factor causing the accumulation of lipids in the cell is nitrogen limitation [18]. Studies performed by Boussiba and Vonshak (1991) showed a simultaneous increase in the content of oleic acid (C18:1 monounsaturated fatty acid) and astaxanthin esters during nitrogen limitation 0.15 g/L and high light amounting 170 *µ*mol photons m^−2^s^−1^ [7]. Furthermore, the results obtained by Damiani et al. (2010) indicate a correlation between the stress conditions of algae growth and the increase in the lipid content in the cell [36]. In this case, the use of unfavorable conditions caused a significant increase in the total lipid when compared to the control culture. In the first variant of stress-conditions, a culture full medium without aeration and continuous light with an intensity of 300 µmoL photons m^−2^s^−1^ were used. In the second variant, a medium without nitrogen and aeration, and the same intensity of light as in the first case were applied. The total lipids content in dry weight was respectively 34.85% and 32.99%. For the control culture with a full medium, continuous aeration with the mixture of air (500–700 mL/min) and CO_2_ (0.3 mL/min) and 12 h of illumination with a white lamp 90 µmoL photons m^−2^s^−1^, the total content of lipids was 15.61%. This study also showed a significant increase in the phospholipid content within cultures maintained in unfavorable conditions. The highest content of neutral lipids and glycolipids was obtained in the first variant of stress conditions and it was 19.80% and 7.85%, respectively, while for the second variant, these values were 16.60% and 6.67%. Only the content of phospholipids was higher in the second variant and was equal to 9.80%, while for the first variant it was 9.5%. In the control culture, the following contents were obtained: neutral lipids 9.20%, glycolipids 3.70% and phospholipids 1.87%. The most common fatty acids in *H. pluvialis* cells are palmitic, linoleic and linolenic acid (Table 1) [19]. The fatty acid profile is dependent on the strain. The *H. pluvialis* KORDI03 profile consists of a low 15.0% content of saturated fatty acids (SFAs) and 6.0% monounsaturated fatty acids (MUFAs), while the content of polyunsaturated fatty acids is 79% [15]. Strain CCALA 1081 isolated from rainwater in Baha Blanca, Argentina, represents a higher SFAs content which ranged from 27.81% to 30.36%, while that of MUFA from 18.96% to 20.07% and that of PUFAs from 43.15% to 47.23% [20]. Cerón et al. (2007) noticed that the nitrogen reduction has a significant impact on the production of fatty acids. Lowering the amount of nitrogen in the culture medium to 1.7 mM led to obtaining 7.60% of fatty acids in dry weight, while the culture maintained in a medium with a higher nitrogen content of 4.7 mM presented a lower amount of fatty acids: 2.1% d.w. In the aforementioned study, it was also demonstrated that the nitrogen content has an effect on the fatty acid profile. With a reduction of nitrogen content to 2 mM, the oleic acid level increases, which is 50% of the total fatty acid content [37]. As for Liang et al. (2015) research, the culture of microalga under control conditions that promote the multiplication of the biomass resulted in a low total lipid content of 13.6%. This research also proved the influence of stress factors on lipid accumulation in the cell. Biomass cultivation in a medium without nitrogen resulted in the accumulation of lipids at the level of 46.71% d.w. In the second variant, an increase in the light intensity from 50 to 350 µmoL photons m^−2^s^−1^ and the use of medium without nitrogen caused an increase in the lipid content to 46.87% d.w. The best results—56.92% d.w.—were obtained for the full medium and a high light intensity of 350 µmoL photons m^−2^ s^−1^ [38].

### 4.3. Carotenoids

The carotenoid content also changes during cell transformation. It increases in *H. pluvialis* cell from 0.5% d.w. in the green phase to 2%–5% d.w. in the red phase. Lutein with a content of 70%–80% is the main carotenoid in green cells. The second component with the highest content is β-carotene (16.70% d.w.), the amounts of violaxanthin and neoxanthin are, respectively, 12.5% and 8.3% d.w. The aforementioned compounds are not found in red phase cells or are only present in small amounts. The next pigment, present only in green cells, is chlorophyll, the content of which is 1.5%–2% d.w. [14]. Astaxanthin is the most important carotenoid obtained from *H. pluvialis* and it is accumulated inside the cell only during the red phase. Its content can reach up to 80%–99% of the total carotenoids [42,43]. According to Web of Science (accessed 3 August 2020), 850 publications were closely related to the topic of the carotenoids, 319 to lipids and 248 to proteins from *H. pluvialis*. Studies on the effects of external factors are mainly based on the accumulation of astaxanthin. However, it is equally important to carry out more thorough research to optimize the culture process in order to increase the content of lipids and proteins. *Haematococcus pluvialis* is a promising cell factory for biofuels and animal feed. Unfortunately, there is not much research concerning the optimization of production and obtaining of triglycerides and astaxanthin simultaneously; obtaining two metabolites of *H. pluvialis* from one culture would make the process more profitable.

## 5. Astaxanthin as a Valuable Biologically Active Compound

Astaxanthin (3,3′-dihydroxy-β,β′-carotene-4,4′-dione) belongs to the group of carotenoids naturally occurring in such organisms as microalgae, crustaceans, fish and some birds [44,45]. This red carotenoid pigment is classified as xanthophyll due to its powerful antioxidant ability. Astaxanthin is made of two β-ionone ring systems within its structure that are linked by a polyene chain and contain the oxygenated keto and hydroxyl moieties [46]. Due to its structure, astaxanthin is a promising factor in the prevention of diseases associated with oxidative stress, including diseases of the vascular and cardiac system, diabetes and cancers [47]. The construction of astaxanthin enables it to combine biological membranes and to reduce and stabilize free radicals. Most often people supply astaxanthin from foods such as seafood. Ambati et al. (2014) noted 6 mg of astaxanthin per kg of flesh of European trout, 25 mg/kg in flesh of Japanese trout and 6–8 mg/kg in flesh of farmed Atlantic salmon [48]. In nature, the highest content of astaxanthin is in microalga *Haematococcus pluvialis* which can accumulate up to 5% d.w. Synthetic astaxanthin dominates commercially because of lower costs of production [49]. *H. pluvialis* is perceived by many researchers as a primary source of astaxanthin for the food industry because of 3*S*, 3*S*’ stereoisomer, which is the most effective isomer for human application, compared to such isomers as 3*R*, 3*S*’ and 3*R*, 3*R*’. This spatial arrangement of atoms increases the bioavailability of astaxanthin [50]. Astaxanthin has a 10 times stronger antioxidant activity than that of β-carotene, and 100 times stronger than that of α-tocopherol [51]. 

### 5.1. Astaxanthin Accumulation

The life cycle of *H. pluvialis* is influenced by inductive factors that cause astaxanthin accumulation and non-inductive factors necessary to maintain cell growth during the green phase [50]. The synthesis of astaxanthin in *H. pluvialis* cell occurs in the red phase. To enter this phase, unfavorable environmental conditions must occur, such as nitrogen reduction, high light intensity, salinity, pH change or extreme temperatures. Ethanol regulates the expression of carotenogenesis genes and significantly increases the accumulation of astaxanthin in cells [52]. Ota and Kawano (2019) have shown a protective activity of astaxanthin on the cell. During short-term exposure of cells to high light intensity (10–15 min), astaxanthin migrated from the inside of the cell to its wall. After reducing the intensity of light, astaxanthin returned to the centre of the cell. This phenomenon indicates that the red pigment contained in the cell is used as a protective factor against high light intensity [16]. Astaxanthin and triacylglycerols (TAGs) accumulate together in the lipid bodies during the red phases. Astaxanthin is synthesized from isoprene units, which are the basic units for carotenoid synthesis [14]. Isopentenyl pyrophosphate (IPP) is the precursor for carotenoid synthesis; isopentenyl is converted to pyrophosphate using isopentenyl pyrophosphate isomerase. Then phytoene synthase catalyzes the phytoene synthesis reaction. The conversion of phytotene to lycopene takes place with the participation of the following enzymes: phytoene desaturase and carotene desaturase. Lycopene β-cyclase is responsible for the cyclization of lycopene to β-carotene. The last step involves the conversion of β-carotene to astaxanthin using the enzymes β-carotene ketolase, β-carotene oxidase and β-carotene hydroxylase [21].

### 5.2. Astaxanthin Recovery

Recovery of astaxanthin from *Haematococcus pluvialis* consists mainly of the following stages: (I) cell breakage, (II) alkaline treatment, (III) solvent extraction, (IV) solvent removal, (V) purification, (VI) resuspension in oil and (VII) single step-alkaline extraction [33,53,54]. Due to the thick cell wall, microalgae require mechanical disruption of the cells before applying the solvent [55]. Mechanical methods consisting of grinding, compression or pressing under high pressure are the most effective. To obtain the highest durability, it is necessary to dry the pigment or all biomass [54]. The use of traditional solvent extraction has many disadvantages, such as large volumes of organic solvents, high extraction temperature, the risk of the thermal degradation of extracted molecules or the presence of solvent residues in the extracts [55]. Therefore, innovative extraction techniques are becoming more and more popular [56,57,58]. Among them, we can distinguish microwave-assisted extraction (MAE) [56], ultrasound-assisted extraction (UAE) [56,59], supercritical fluid extraction (SFE) [55,60,61] and enzyme-assisted extraction (EAE). The main advantages of those methods are reduced solvent usage, short extraction time and higher extraction yield [58].

In the case of MAE, the extraction induces changes in the cell wall caused by electromagnetic waves. The MAE extraction rate can be correlated with heat and mass transfer gradients working in the same direction [62]. In the study published by Ruen-ngam et al. (2011), a 74% recovery rate of astaxanthin was reached using the MAE method with acetone, for a duration of 5 min at 75 °C [56]. Ultrasound assisted extraction is recognized as an alternative to traditional astaxanthin extraction methods. In addition, ultrasound procedures are much faster than traditional methods [63]. Ruen-ngam et al. (2011) obtained the highest astaxanthin recovery (73%) after 60 min of extraction with acetone at a temperature of 45 °C [56]. Di Sanzo et al. (2018) showed that SFE was very efficient in the recovery of astaxanthin; as with optimal extraction conditions (50 °C and 550 bars), a 98.6% recovery rate was achieved [61]. Wang et al. (2012) showed similar results with the astaxanthin yield being 87.4% under optimal experimental conditions (65 °C, 435 bar, co-solvent 2.3 mL/g) [60], whereas Molino et al. (2018) observed 92% for 65 °C and 550 bar [55]. A promising technique is enzyme-assisted extraction, where optimal experimental conditions (pH, temperature and time) and properly selected enzymes (mainly pectinase, cellulase) and their doses allow for efficient astaxanthin release from *Haematococcus pluvialis*. In the case of cellulase used in a 3% concentration, the extraction yield was about 60% (pH 5, 3 h, 65 °C) [64]. It shows that innovative extraction techniques provide a high extraction yield of this pigment. Obtaining astaxanthin from microalgae is an expensive process due to the cost of biomass cultivation, which accounts for 20%–30% of the total production costs [55].

According to the Web of Science database (accessed 3 August, 2020), 268 publications on extraction of astaxanthin from *Haematococcus pluvialis* have been published so far. A total of 98 publications concerned the traditional solvent extraction, 52–SFE, 12–UAE, 11–MAE and 3–EAE. These data also show a recent growing interest in modern extraction techniques: 43 publications on SFE have been published in the recent 10 years.

## 6. Biological Properties of Astaxanthin

Antioxidants are chemical compounds that prevent oxidation in small concentrations or delay the oxidation of substrates [33]. The term “antioxidants” also includes some semi-synthetic analogues of plant substances, natural plant extracts, synthetic food additives and medicines. Antioxidative compounds are divided into (I) enzymes (superoxide dismutase, catalase and glutathione peroxidase) and (II) non-enzymatic substances (vitamins A, C, E, carotenoids, polyphenols and glutathione). Oxidative damage is caused by reactive oxygen species (ROS) and free radicals [52]. Free radicals are molecules containing at least one unpaired electron on the outer electron shell. As a consequence, free radicals seek to pair electrons either by taking them away or giving them to other molecules. In the body, around 90% of free radicals are generated by the respiratory chain, while the remaining 10% originates from physiological reactions of the cell [65]. 

The formation of free radicals can also be caused by ultraviolet, ionizing radiation, ultrasound or elevated temperature and in the metabolism processes of various exogenous chemical compounds [65,66]. Importantly, free radicals are necessary for the proper course of many life processes. They partake in the regulation of gene expression, protein phosphorylation and calcium concentration in cells, activate control of proteins cell divisions, and participate in the elimination of microorganisms. However, the occurrence of excessive free radicals can lead to structure damage and the disturbances of vital cell functions, disruption of homeostasis and even death as a result of apoptosis or necrosis [66,67]. Carotenoids have a polyene chain and long conjugated double bonds. The structure of these compounds is responsible for antioxidant activity, such as the hardening of singlet oxygen and the removal of radicals to complete the chain reactions [49]. The structure of astaxanthin consists of a conjugated polyene chain in the center and hydroxyl and ketone moieties on each ion ring. Astaxanthin is characterized by higher biological activity than other antioxidants because it can bind to the cell membrane from the inside to the outside [68]. The astaxanthin end ring captures radicals on the surface and inside the cell membrane, while the polyene chain does so only in the cell membrane [69]. Astaxanthin, after the quenching of singlet oxygen, dissipates energy through interaction with the solvent; then, the carotenoid structure returns to its original state [46,70]. According to the reviewed studies, astaxanthin exhibits higher antioxidant activity compared to various carotenoids, such as α-carotene, β-carotene, lycopene and lutein; additionally, it induces paroxidanase, an enzyme with antioxidant activity [70,71]. Miki (1991) found out that astaxanthin had 10 times higher antioxidative activity than lutein, zeaxanthin and β-carotene canthaxanthin, and 100 times higher than that of *α*-tocopherol [51].

### 6.1. Anti-Lipid Peroxidation Properties

Lipid peroxidation involves the oxidation of lipids and in consequence formation of lipid peroxides. The reaction consists of three stages: initiation, propagation and termination. Lipid peroxidation yields aldehydes (e.g., malondialdehyde-MDA), hydroxyaldehydes (e.g., 4-hydroxynonenal) and hydrocarbons (e.g., ethane) [72]. These compounds can modify the physical properties of cell membranes, including reduction in the hydrophobicity of the lipid interior of the membranes, disturbance in the lipid asymmetry of the membranes, inhibition of the activity of transporting proteins, depolarization of membranes and inhibition of the activity of membrane enzymes. These changes may result in the loss of intracellular membrane and plasma membrane integrity [73]. The antioxidant activity of astaxanthin is pH-dependent. Mano et al. (2018) showed that astaxanthin strongly inhibits the formation of by-products of lipid peroxidation (thiobarbituric acid reactive substances) in zwitterionic phosphatidylcholine liposomes at pH 7.4 (80%) and at pH 8 (65%). Furthermore, it also slightly inhibits the process of lipid peroxidation at pH 6.2 (20%) and 6.8 (30%) [74]. In the case of ulcerated rats, astaxanthin supplementation decreased the anti-lipid peroxidation effect. The level of thiobarbituric acid reactive substances in serum (as nmoL malondialdehyde per mg serum) decreased from 3.76 nmoL MDA/mg (control) to 2.04 nmoL MDA/mg, 1.94 nmoL MDA/mg and 1.56 nmoL MDA/mg, respectively, for a dose of astaxanthin 100, 250 and 500 µg/kg b.w per day [75].

Interestingly, astaxanthin is more effective than β-carotene in the prevention of lipid peroxidation. As Goto et al. (2001) found out, astaxanthin was twice as effective as β-carotene in inhibiting ADP and Fe^2+^ induced liposomal peroxidation. The supposed mechanism of astaxanthin activity in the prevention of lipid peroxidation results from the interaction of its ending rings of with hydrophilic polar sites of phospholipids membrane and forming an intramolecular hydrogen-bonded five-membered ring which increases the hydrophobicity of astaxanthin [45]. The effect of astaxanthin on the lipid peroxidation was assessed in LDL studies in ex vivo conditions [76]. For two weeks, volunteers were given different doses of astaxanthin (1.8, 3.6, 14.4 and 21.6 mg/day). Samples of LDL from the group of people who received astaxanthin were characterized by lower susceptibility to oxidation when compared to the control group (LDL from group not consuming astaxanthin) [77].

### 6.2. Anti-Inflammatory Effects

Chronic inflammation is the main pathophysiological factor in many diseases, such as diabetes or many neurodegenerative diseases. Due to the high percentage of polyunsaturated fatty acids in the plasma membranes, immune cells are particularly sensitive to oxidative stress, overproduction of reactive oxygen species disturbs the antioxidant balance [78]. Astaxanthin is a powerful antioxidant that inhibits inflammation in biological systems. This carotenoid is able to regulate the immune response or reduce inflammation associated with peripheral diseases [79,80]. Studies have shown that astaxanthin can regulate microglial cells, which are non-neuronal cells of the central nervous system. Microglial cells are tissue-specific resident macrophages which control homeostasis and are involved in the immune response. These cells after recognizing the threat, such as the presence of pathogens or cell damage, release pro-inflammatory cytokines such as IL-1β, TNF-*α*, IL-6 and NO. In the initial phase, this response is effective in danger neutralization; however, due to the toxic nature of proinflammatory molecules, prolonged action of microglia can destroy the central nervous system [81,82]. In studies of Choi et al. (2008) and Kim et al. (2010), the use of astaxanthin resulted in decreased secretion of IL-6, Cox-2 and iNos/nitric oxide in microglia during the presence of bacteria [83,84]. Park et al. (2009) reported a reduction of κB and neurodegeneration in the frontal cortex and hippocampus. This research described the performance of mice treated with astaxanthin in the Morris water maze [85]. Astaxanthin plays important roles in the amelioration of inflammatory diseases including arteriosclerosis, inflammatory bowel disease, sepsis, rheumatoid arthritis, gastric inflammation and brain inflammatory diseases.

### 6.3. Anti–Diabetic Activity

**Figure 4:**
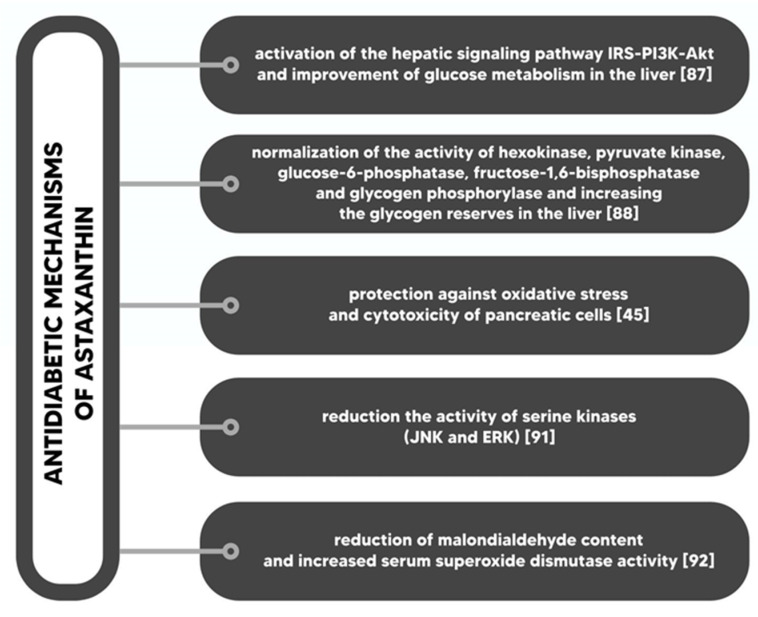
Antidiabetic mechanisms of astaxanthin.

Insulin resistance is a serious disorder of glucose homeostasis, which is characterized by decreased insulin sensitivity of various tissues, such as skeletal muscle, adipose tissue or liver. Insulin resistance is one of the causes of type 2 diabetes and gestational diabetes; usually, it is also a factor in the course of type 1 diabetes. Insulin resistance is often accompanied by hyperinsulinaemia [86]. Astaxanthin improves the whole body’s insulin sensitivity and insulin stimulated glucose uptake in the muscle of insulin-resistant animals. Astaxanthin reduces the level of oxidative stress caused by hyperglycemia in pancreatic β cells and has a positive effect on serum glucose and insulin [87]. Studies in astaxanthin-fed mice have reported increased insulin sensitivity in both hypertensive rats and mice fed with high fat and high fructose diets, while the level of albumin in the urine of diabetic mice was significantly lower than in the control group (without astaxanthin in diet) [87,88]. Bhuvaneswari et al. (2012) confirmed that astaxanthin stimulates the signaling pathway of the insulin receptor substrate (IRS) -PI3K-AKT, due to the reduction of the serine phosphorylation of IRS proteins, and increases glucose metabolism by regulating metabolic enzymes [89]. Astaxanthin is also responsible for lowering the level of cholesterol in blood as well as the level of triglycerol in the liver and stimulating the expression of antioxidant genes. In addition, astaxanthin reduces the expression of CYP2E1 and, as a result, increases the sensitivity of cells to insulin and inhibits liver damage [90]. In the early stages of diabetes, astaxanthin protects pancreatic β cells, increases insulin sensitivity and improves glucose metabolism. As a consequence, insulin resistance and blood glucose levels decrease. Supplementation with astaxanthin reduces oxidative stress, inflammation and lipid peroxidation, therefore it prevents such complications of diabetes as: retinopathy, neuropathy, nephropathy and cardiovascular complications [91]. Mechanisms underlying antidiabetic effects of astaxanthin (Figure 4) are as follows: (I) activation of IRS-PI3K-Akt signals and increased glucose metabolism in the liver; (II) normalization of hexokinase activity, pyruvate kinase, glucose-6-phosphatase, fructose-1,6-bisphosphatase and glycogen phosphorylase; (III) protection against oxidative stress and cytotoxicity in pancreatic cells; (IV) reduction of serine kinases activity; and (V) reduction of MDA [47,89,92,93,94].

### 6.4. Anticancer Activity

Astaxanthin has the ability to inhibit cancer cell growth. Antioxidant compounds decrease mutagenesis and carcinogenesis by inhibiting oxidative damage to cells. Studies have reported that astaxanthin not only inhibits the proliferation of colon cancer cells but can also cause their apoptosis. Palloza et al. (2009) used an extract from *H. pluvialis*, which inhibited the growth of human colon cancer cells. Astaxanthin was included in the extract and was responsible for stopping the progression of the cell cycle and promoting the apoptosis [95]. Astaxanthin showed higher antitumor activity than other carotenoids, including canthaxanthin and β-carotene [96,97]. Jyonouchi et al. (2008) and Nakano et al. (2008) proved increased levels of immune cells, natural killer cells and plasma γ interferon in mice after astaxanthin treatment [98,99]. The oral administration of astaxanthin inhibited carcinogenesis in the urinary bladder of mice and in the colon and oral cavity of rats. This effect has been partially assigned to the suppression of cell proliferation. Research performed by Song et al. (2011) showed high anti-proliferative activity of astaxanthin against tumor cells like: SHZ-88 breast cancer cells, hepatoma CBRH-7919 cells and Lewis cells. A significant correlation was observed between astaxanthin concentrations and anti-proliferative activity. The most sensitive cell line to astaxanthin with half a maximal inhibitory concentration of 39 μM was the CBRH-7919 line [100]. 

According to Web of Science (accessed on 3 August 2020), 269 publications refer to anti-inflammatory effect of astaxanthin and 238 publications refer to anti-lipid peroxidation effect. Studies on the effects of astaxanthin on human health mainly cover such disorders as metabolic diseases, cancer and inflammatory diseases, as well as skin and eye conditions. Studies on the effects of astaxanthin on animal organisms are promising, mainly due to the antioxidant properties of the compound. Research has demonstrated an astaxanthin-induced reduction in blood glucose and insulin levels; astaxanthin has also sensitized cellular receptors to insulin. Correlation of excess free radicals and the development of metabolic diseases, such as type 2 diabetes and insulin resistance, have been confirmed by many studies. However, not all of them prove the antioxidant effect of carotenoids, which necessitates the use of new models and pathways.

### 6.5. Other Potential Applications of Astaxanthin

Astaxanthin has a beneficial effect, due to its high antioxidant and anti-inflammatory potential. As a result, more and more research is being conducted on the biological activities of astaxanthin in the context of the nervous, visual and cardiovascular systems.

Hypertension is a major risk factor for cardiovascular disease. Overproduction of reactive oxygen and nitrogen species results in diseases, such as hypertension, atherosclerosis, endothelial dysfunction or arrhythmias [68]. Oral administration of astaxanthin to hypertensive rats decreased nitric oxide products and lowered blood pressure [101,102]. In vivo studies have shown that astaxanthin supplementation decreased the level of TG, TC, LDL-C, IL-6, CRP and LPO; as a result, this improved the antioxidant defense capacity and choroidal blood flow velocity. Astaxanthin also increased SOD activity and decreased PG-E2, LT-B4, NO, IL-8 and IFN- γ production [103,104,105]. Astaxanthin has a cardiovascular protective effect in animals, but there is a lack of research supporting the therapeutic benefit of astaxanthin in atherosclerotic cardiovascular disease in humans.

Astaxanthin has a beneficial effect in the prevention and treatment of eye diseases, such as age-related macular degeneration, glaucoma, cataract or keratopathy. Astaxanthin crosses the barrier of the circulatory system and the retina of eye. As the only antioxidant, it builds into the eye’s cell membrane, protecting it against damage and free radicals. Astaxanthin due to antioxidant activity inhibited ischemia induced retinal cell death [106]. Supplementation of rats with astaxanthin at a dose of 5 mg/kg/day for 8 weeks resulted in a decrease in retinal apoptosis, a decrease in the production of protein carbonyl and NOS-2, which brought an increase in retinoprotective properties [107]. Astaxanthin counteracts many eye diseases because of its anti-inflammatory, antioxidant properties and regulation of metabolism [108]. 

Fakhri et al. (2019) showed that astaxanthin blocks neurodegenerative pathways, such as oxidative stress, inflammation and apoptosis, and can pass through the blood brain barrier [109]. Supplementation lowers the expression of Bax and Cleaved-caspase-3, which inhibits and reduces neuronal apoptosis and pathological tissue damage. Oral supplementation with astaxanthin in rats after surgery decreased the expression of NF-KB and TNF-α, which resulted in a reduction of cerebral edema and neurological dysfunction [110,111]. Astaxanthin is considered as a multi-target pharmacological agent against neurological disorders including Parkinson’s disease, Alzheimer’s disease, brain and spinal cord injuries, neuropathic pain, aging, depression and autism [112].

## 7. Astaxanthin in the Human and Animal Diet

Astaxanthin is commercially available mainly in the form of dietary supplements, oils or dried aplanospores. The cell walls of aplanspor must be broken because they undergo partial digestion. Oils containing astaxanthin are not organoleptically attractive, due to the smell and taste of algae [113]. Satisfactorily, in recent years, there has been more and more research into the incorporation of astaxanthin into food and animal feed. 

### 7.1. Human Diet

Good results have been obtained for whole grain cakes. In vitro digestion studies showed a decrease in glucose release proportional to the rise in astaxanthin concentrations in the cake formulation [114]. Studies performed by Mercke et al. (2003) confirmed the beneficial effect of lipids on the absorption of astaxanthin. Patients took 40 mg of astaxanthin in various oil-based formulations. All formulation carriers increased the absorption of astaxanthin. The best effect was demonstrated by formulations with the highest content of the hydrophilic synthetic surfactant [115]. Furthermore, consumption of *H. pluvialis* biomass as a dietary supplement in combination with olive increases the antioxidant properties and bioavailability of astaxanthin [116]. In the stomach, astaxanthin accumulates in drops of lipids and is incorporated into micelles that diffuse into plasma membranes of enterocytes and are transported in the circulation by low-density lipoprotein (LDL) and high-density lipoprotein (HDL) [117]. Astaxanthin in combination with fish oils increases hypolipidemic/hypocholesterolemic effect in plasma. The combination showed increased phagocytic activity of activated neutrophils [118]. Diet and smoking have a big influence on the absorption of astaxanthin. The bioavailability of astaxanthin in smokers is reduced by 40% [119]. Carotenoids are absorbed into the bloodstream in a similar manner as lipids; subsequently, they are transported via the lymphatic system to the liver. The absorption process highly depends on the accompanying dietary components. The intake of carotenoids can be increased by a high cholesterol diet, while a low-fat diet reduces their absorption [48]. Carotenoid absorption involves the following steps: (I) release of carotenoids from food matrix, (II) solubilization of carotenoids into mixed lipid micelles; (II) cellular uptake of carotenoids by intestinal absorptive cells (enterocytes); (IV) incorporation of carotenoids into chylomicrons; (V) secretion of carotenoids and their metabolites associated with chylomicrons into the lymph within the systemic circulation; and (VI) tissue distribution, metabolism and recycling of carotenoids [76,120,121,122,123]. Astaxanthin is well tolerated by the human organism and numerous studies have not revealed any toxic effects [124,125]. The European Food Safety Authority (EFSA) on Additives and Products or Substances used in Animal Feed (FEEDAP) recommended the use of astaxanthin in a dose of 0.034 mg/kg of body weight, 2.38 mg per day in a 70 kg human [126,127]. According to Spiller and Dewell (2003), a daily dose of astaxanthin equal to 2–4 mg is safe. They did not report toxic effects when adults consumed up to 6 mg/day. Satisfactory astaxanthin bioavailability results were obtained with a daily astaxanthin dose of 40 mg/day. This dose was well tolerated by the human body [115]. Commercial astaxanthin-containing products are available in the form of both daily capsules, soft gels, energy drinks and powders [48]. Research into patients suffering from functional dyspepsia indicates that safety doses are from 16 to 40 mg per day [128]. The presence of dietary fat enhances the assimilation of astaxanthin in the small intestine [119]. Excessive consumption of astaxanthin leads to the accumulation of pigment in tissues and skin, which is desirable in the breeding of some animals, e.g., salmon. Studies on rats showed an increase in the content of antioxidant enzymes, such as catalase, superoxide dismutase and glutathione peroxidase after cyclic dosing of astaxanthin [116,124]. Astaxanthin has the status of pure antioxidant, which means that it does not have any pro-oxidative properties [129]. The main obstacle to the industrial use of astaxanthin is its low chemical stability during storage. To maintain its high stability, it is necessary to provide protection against adverse effects of temperature, pH and light exposure [48]. The best solution for achieving high bioavailability and stability is combining astaxanthin with edible oils. According to Ranga Rao et al. (2007), astaxanthin was fairly stable in rice bran, coconut, groundnut, mustard, gingelly, palm oils, sunflower and olive, when stored at room temperature for four months [130]. Ambati et al. (2014) showed the stability of astaxanthin at 70–90 °C in ricebran, palm oils and gingelly with an 84%–90% retention of the astaxanthin content. Compounds such as polyphenols, tocopherols or flavonoids have a positive impact on improving the stability of carotenoids [48]. According to Peng et al. (2010), encapsulation of astaxanthin within liposomes improved stability and bioavailability [118]. Similar effects were observed using microencapsulation with polymeric nanospheres, b-cyclodextrin, hydroxypropyl-b-cyclodextrin and sulfobutyl ether b-cyclodextrin, as documented by various researchers [48,131,132]. Satisfactory results have been obtained for the spray drying of microcapsules with the addition of maltodextrin and gelatin in a ratio of 2.1:1. The microcapsules showed good spherical shapes with a smooth surface and good solubility. The use of cheap carriers makes the process economically profitable [129].

### 7.2. Animal Feed

Astaxanthin has been used in feeding animals due to its strong antioxidant activity and safety of use and also because this pigment improves the organoleptic properties of animal products. Animals cannot synthesize carotenoids on their own, which is why they must ultimately obtain this pigment from the diet, in which plants and algae are rich [133,134]. 

Astaxanthin from *Haematococcus pluvialis* is mainly used in the aquaculture industry for the pigmentation of fish [14]. This interest in natural pigments results from the growing awareness of consumers and their demand for natural products [31,123]. Astaxanthin improves not only the coloration of many aquatic animal species, but also increases the survival of animals, their stress tolerance, disease resistance and growth performance [123]. The intensity of the pink-orange color of animals such as lobsters, aquarium fish, shrimps and salmon increased after astaxanthin supplementation. In the literature, there are examples of a beneficial effect of this natural pigment on animal health and quality of animal products. Astaxanthin supplemented to the diet of Atlantic cod (*Gadus morhua* L.) increased the fertilization, improved survival of larvae and reduced the embryonic mortality [135]. Dore and Cysewski (2003) found that natural astaxanthin from *Haematococcus* was equally effective with synthetic astaxanthin at pigmenting rainbow trout, when used at a dose of 50 mg/kg of feed. What is important it that the *Haematococcus* algae meal as a source of astaxanthin is safe and non-toxic [31]. It was found that *Haematococcus pluvialis* supplemented to the diet of rainbow trout (3 g/kg of feed) effectively enhanced the antioxidant activity and some biochemical parameters (e.g., decrease in serum glucose levels, decrease in triglyceride and cholesterol levels) [136]. Astaxanthin is of great interest in crustacean farming, such as the giant tiger prawn (*Penaeus monodon*). This carotenoid reverses the blue color syndrome found in farmed prawns with pigment deficiency. Food containing 50–100 g of astaxanthin per 1 kg of feed restored and harmonized pigmentation of shrimps within 4 weeks [137]. Ju et al. (2012) additionally found that the defatted *Haematococcus pluvialis* meal can serve as not only an alternative pigmentation ingredient in Pacific white shrimp feed, but also as a source of protein. This additive caused additionally a higher growth rate [138]. 

*Haematococcus pluvialis* and its pigment—astaxanthin—can also be used in livestock feeding. Astaxanthin used as a feed additive in poultry farming strengthened muscles, enhanced yolk color and prevented fat oxidation. The addition of this carotenoid did not, however, affect egg weight or size [139]. Astaxanthin improved yellow pigmentation of the feet and beaks of poultry. Chickens fed with astaxanthin-containing feed not only increased their weight faster and had a greater increase in the muscle mass, but also showed increased fertility [140]. Supplementation with astaxanthin reduced chicken mortality associated with yolk sac inflammation and further increased resistance to *Salmonella* infection [141]. Waldenstedt et al. (2003) showed that astaxanthin-rich algal meal (7, 36, or 179 mg astaxanthin/kg feed) reduced caecal colonization of *Clostridium perfringens* of broiler chickens. The concentrations of this pigment increased in the kidney, intestine and breast muscle when compared to the control birds [142].

One of the most important features of pork for the consumer is its color. Oxymyoglobin oxidation is the main cause of meat color deterioration. Smith et al. (2003) proved that vitamin E added to the feed has an antioxidant effect and extends the usefulness of pork [143]. Astaxanthin accumulated in muscle tissue as a result of feeding has a better antioxidant effect than that added during meat processing. This is a very promising result, so much so that astaxanthin’s antioxidant activity is four times higher than that of Vitamin E. Antioxidants not only affect the quality of meat, but also the health and condition of pigs [144]. Yang et al. (2006) demonstrated a tenfold reduction in back fat content and an increase in muscle mass after 14 days of feeding with 3 mg/kg of astaxanthin. Further reports refer to the improvement of pork carcass quality after using 48 mg/kg of astaxanthin for 3 months and 66.7 mg/kg for 42 days [139,145]. The supplementation of astaxanthin to the pig diet increased also the color of meat. Loin chops from pigs supplemented with dietary astaxanthin were darker and less yellow than loin chops from control pigs [146]. Furthermore, Bergstrom et al. (2009) found out that the loin muscle of pigs fed with astaxanthin (5 and 10 mg/kg) had a darker color, which can contribute to improved consumer acceptance of fresh pork [141].

Serwotka-Suszczak et al. (2019) showed that the extract from *Haematococcus pluvialis* additionally enriched with Mg(II) ions during cultivation improved insulin resistance in equine adipose-derived stromal cells. Therefore, this microalga can be used in the treatment of metabolic disorders [147]. Astaxanthin supplemented to the diet of dogs exerted its antioxidant properties, increased cell-mediated and humoral immune response and reduced DNA damage and inflammation [80].

There are several commercially available products based on *H. pluvialis*-derived astaxanthin, such as AstaEquus^®^ (astaxanthin extract feed supplement for horses) and Novaasta^®^ (astaxanthin extract feed supplement for animals): both produced by BioReaI, Sweden; Asta powder™ (powder for animal feed supplement, Atacama Bio Natural, Chile); Naturose^TM^ (Algae meal; pigmentation source for ornamental fish and animals, produced by Cyanotech Corporation, USA) [13,14,31]; and astraZanthin^TM^ (astaxanthin for dogs, LaHaye Laboratories, Redmond, WA, USA) [80].

## 8. Conclusions

Microalga *H. pluvialis* is the most attractive natural source of astaxanthin because it has the highest content in dry weight. The production of *H. pluvialis* on an industrial scale still faces difficulties related to the sensitivity of the strain to changes in growth conditions. Improving astaxanthin accumulation by *H. pluvialis* would reduce production costs and increase the availability of this carotenoid. The properties of astaxanthin, such as antioxidative, anti-inflammatory, antineoplastic, immunomodulatory activity and safety of its use, resulted in the increased interest in astaxanthin on the part of many scientific centers and food producers. Many scientific reports indicate that astaxanthin has a beneficial effect on the human body. There is a need to deepen the knowledge about astaxanthin. Some tests still need validating before it is possible to explain exactly the processes behind the effects of astaxanthin.

## Figures and Tables

**Table 1:** Comparison of fatty acid compositions (%) of different *H. pluvialis* strains.

Fatty Acids	Kim et al., 2015 [19]	Lorenz, 1999 [39]	Scodelaro Bilbao et al., 2016 [40]	Lei et al. 2012 [41]
C12:0 lauric	N/A	0.1	N/A	0.28
C14:0 myristic	0.1	0.5	1.99	0.65
C15:0 pentadecanoic acid	0.1	N/A	N/A	0.25
C16:0 palmitic	13.7	29	22.9	12.7
C16:1 palmitoleic	0.5	0.6	0.35	0.7
C16:2	0.4	N/A	N/A	N/A
C16:3	3.5	N/A	N/A	N/A
C16:4	3.3	N/A	N/A	N/A
C17:0 margaric	N/A	0.2	N/A	0.23
C17:1 margaroleic	N/A	1.3	N/A	0.0
C18:0 stearic	0.7	2.1	1.15	4.79
C18:1 oleic	4.9	25.9	16.3	11.2
C18:2 linoleic	24.9	20.8	23.9	13.0
C18:3 linolenic	39.7	12.8	12.5	2.84
C18:4 octadecatetraenoic	5.8	1.4	N/A	N/A
C20:0 arachidic	N/A	0.6	N/A	0.35
C20:1 gadoleic	0.5	0.3	N/A	1.3
C20:2 eicosadenoic	N/A	1.2	2.21	0.87
C20:3 eicosatrienoic gamma	N/A	0.5	N/A	0.18
C20:4 arachidonic	0.9	1.4	1.92	1.77
C20:5 eicosapentaenoic	0.6	0.6	0.66	0.99
C22:0 behenic	N/A	0.4	N/A	0.16
C24:0 lignoceric	0.3	0.2	0.33	0.4
C24:1 nervonic acid	0.1	0.1	N/A	0.14
Σ SFAs	15	33.2	25.3	19.8
Σ ΜUFAs	6	28.1	16.6	12.97
Σ PUFAs	79.1	38.7	41.2	19.65

N/A–not available.
